# Expression Levels of Three Key Genes CCNB1, CDC20, and CENPF in HCC Are Associated With Antitumor Immunity

**DOI:** 10.3389/fonc.2021.738841

**Published:** 2021-09-30

**Authors:** Tengfei Si, Zhenlin Huang, Yuanhang Jiang, Abigail Walker-Jacobs, Shaqira Gill, Robert Hegarty, Mohammad Hamza, Shirin Elizabeth Khorsandi, Wayel Jassem, Nigel Heaton, Yun Ma

**Affiliations:** ^1^ Institute of Liver Studies, King’s College Hospital, Department of Inflammation Biology, Faculty of Life Sciences & Medicine, King’s College London, London, United Kingdom; ^2^ The Ministry of Education (MOE) Key Laboratory for Standardization of Chinese Medicine, Institute of Chinese Materia Medica, Shanghai University of Traditional Chinese Medicine, Shanghai, China; ^3^ Pediatric Liver, GI and Nutrition Centre, King’s College Hospital, London, United Kingdom; ^4^ Transplant Service, King’s College Hospital, London, United Kingdom; ^5^ The Roger Williams Institute of Hepatology, Foundation for Liver Research, London, United Kingdom

**Keywords:** HCC, prognosis, antitumor immunity, inhibitory checkpoint, hepatocarcinogenesis

## Abstract

**Introduction:**

Hepatocellular carcinoma (HCC) is the most common primary liver cancer with a low 5-year survival rate. The heterogeneity of HCC makes monotherapy unlikely. The development of diagnostic programs and new treatments targeting common genetic events in the carcinogenic process are providing further insights into the management of HCC. The aim of this study was firstly to validate key genes that are involved in promoting HCC development and as biomarkers for early diagnosis and, secondly, to define their links with antitumor immunity including inhibitory checkpoints.

**Methods:**

Multiple databases including Gene Expression Omnibus (GEO), Gene Expression Profiling Interactive Analysis (GEPIA), Kaplan–Meier Plotter, UALCAN, and Oncomine were used for target gene screening and establishment of a co-expression network. Clinical data and RNAseq of 367 HCC patients were downloaded from the Cancer Genome Atlas (TCGA) database. The diagnostic and prognostic value of screened genes were tested by receiver operating characteristic (ROC) curve and correlation analysis. The links with the key genes in HCC and antitumor immunity were defined using both blood and liver tissue collected prospectively from HCC patients in our center.

**Results:**

Upregulation of CCNB1, CDC20, and CENPF was commonly observed in HCC and are involved in the p53 signal pathway. The hepatic mRNA expression levels of these three genes were strongly associated with patients’ prognosis and expressed high value of area under the ROC curve (AUC). Further analysis revealed that these three genes were positively correlated with the gene expression levels of IFN-γ, TNF-α, and IL-17 in peripheral blood. In addition, the expression of CENPF showed positive correlation with the percentage of CD8^pos^ T cells and negative correlation with the percentage of CD4^pos^ T cells in the peripheral blood. In the HCC microenvironment, the transcript levels of these three genes and inhibitory checkpoint molecules including PD-1, CTLA-4, and TIM-3 were positively correlated.

**Conclusion:**

The upregulation of CCNB1, CDC20, and CENPF genes was a common event in hepatocarcinogenesis. Expression levels of CCNB1, CDC20, and CENPF showed potential for early diagnosis and prediction of prognosis in HCC patients. There is a close association between three genes and Th1/Th17 cytokines as well as the count of CD4^pos^ and CD8^pos^ T cells. The positive correlation between the three genes and inhibitory checkpoint genes, PD-1, CTLA-4, and TIM-3, indicates that these genes are linked with weakened antitumor immunity in HCC. Our findings may provide further insights into developing novel therapies for HCC.

## Introduction

Hepatocellular carcinoma (HCC) represents more than 90% of primary liver cancers and is a global health problem (1). It is ranked as the fourth leading cause of cancer-related death in the world with a growing incidence (2). Surgery including resection and liver transplantation remains the most effective treatment and could achieve a 5-year survival of 60% to 80%. HCC is usually asymptomatic in the early stage. At diagnosis, many patients have advanced disease with limited treatment options.

There is increasing evidence that genetic alterations play an important role in the development of HCC (3). With the advent of deep sequencing technology, increasing information regarding genetic mutations in HCC has identified several important pathways related to cancer formation (4–7). Attention is being focused on genes involved in key events in hepatocarcinogenesis, such p53, which has been identified as the most frequently mutated tumor suppressor gene in HCC. Research on p53 gene mutations has provided models for developing clinical treatments for HCC.

To date, several specific markers and key pathways involved in the HCC development have been identified potentially to assist early diagnosis and to predict prognosis. Although immunoregulatory therapies such as anti-PD-1 and CAR-T have made progresses in the treatment of solid tumors, the anti-PD-1/CTLA-4 therapy in HCC appears as a challenge. We aim to identify the key genes in the HCC environment, which are associated with the antitumor immunity including adaptive immunity, such as Th1/Th17, and immunoregulation with inhibitory checkpoints.

We conducted an analysis to screen gene markers related to the development of HCC. Using clinical data and RNA-seq (TCGA open-source data) from 367 HCC patients to confirm their diagnostic and prognostic value. In addition, we defined the relationship of these genes with anti-tumor immunity represented by the transcript levels of IFN-γ, TNF-α, and IL-17, the portion of T cell subsets in peripheral blood, and the expression level of inhibitory checkpoint molecules: PD-1, CTLA-4, and TIM-3 in HCC tumor tissue, as well as PD-1 plasma level in peripheral blood.

## Materials and Methods

### Common Differentially Expressed Genes Screening

Four GSE profiles (7–10) from platform GPL570 ([HG-U133_Plus_2] Affymetrix Human Genome U133 Plus 2.0 Array) were used to select differentially expressed genes (DEGs) between normal liver tissue (from healthy donor) and HCC cancer tissue (11). The condition was set at |log FC| > 2, *p*-value < 0.05. All DEGs were uploaded to Venn Diagram online software (http://bioinformatics.psb.ugent.be/webtools/Venn/) to detect commonly co-expressed genes.

### Central Cluster Selection and Enrichment Analysis

The STRING online database (available at https://string-db.org/) was used to detect the functional PPI networks (12). Central cluster from the PPI network was identified through MCODE plug-in of Cytoscape (Node Score ≥ 2, K-Core Value ≥ 2, Max Depth = 100) (13). Genes of central cluster were submitted to the Kaplan–Meier Plotter (https://kmplot.com/analysis/) (14). Liver cancer database was chosen to detect poor prognosis-associated genes (lower overall survival) in HCC patients (log rank *p*-value < 0.05, FDR ≤ 5%). Gene Expression Profiling Interactive analysis (GEPIA) (15) online database (http://gepia.cancer-pku.cn/) was used to further validate the expression of survival-related genes.

Gene Ontology (GO) enrichment analysis was conducted on three main functions: Biological Process (BP), Molecular Function (MF), and Cellular Component (CC). For the Kyoto Encyclopedia of Genes and Genomes (KEGG) pathway analysis, ClueGo in Cytoscape was used to perform single cluster analysis. Bonferroni step down method was used by default for multiple testing correction. Only results with a *p*-value < 0.05 and false discovery rate (FDR) < 5% were selected (16). Genes commonly involved in the enriched pathways were screened as core genes.

### Expression and Correlation Network of Core Genes

UALCAN (http://ualcan.path.uab.edu) (17), GEPIA and Oncomine (https://www.oncomine.org) (18) databases were searched for the relative expression of core genes across HCC and normal liver tissue, as well as in different tumor sub-groups based on tumor stages (AJCC-TNM), tumor grade (histology), or other settings. The correlations between core genes and cancer immune infiltrates were investigated *via* Tumor Immune Estimation Resource (TIMER) (http://timer.compgenomics.org/) (19). Principal component analysis (PCA) was performed to compare the distribution differences between LIHC tumor, LIHC normal (HCC adjacent non-tumor liver), and donors’ normal liver tissue on the expression of core genes. Raw data downloaded from TCGA database and processing were conducted by R studio software (version 3.6.1; R studio, Boston, Massachusetts); RNAseq and related clinical data of a total of 367 tumor tissues and 50 normal liver tissues were used to construct a co-expression network.

### Validation Cohort: Patients and Sample Collection

Blood samples were accessed *via* King’s College Hospital, Liver Biobank (*n* = 50). Non-identifying clinical features are summarized in **Table 1**. Patients were stratified into three groups according to whether having received treatment including loco-regional therapy (microwave ablation, chemoembolization), radiation therapy, chemo-/biological therapy, immunotherapy, and surgery as well as tumor status before sample collection: (1) untreated group (*n* = 30), (2) treated HCC with active/residual tumor (*n* = 8), and (3) treated HCC without active/residual tumor (*n* = 12). Peripheral blood mononuclear cells (PBMCs) were isolated using density gradient centrifugation technique (20). Sixteen paired tumor tissue and adjacent liver tissue, as well as 12 normal liver tissue samples from patients with hemangioma or focal nodular hyperplasia (FNH) were accessed *via* the Liver Biobank, Institute of Liver Studies, King’s College Hospital (REC NOS). This study was approved by the Ethics Committee of King’s College Hospital (Ethic REC No. 15/LO/0363, IRAS No.169524) and full consent was obtained from each participant before blood and liver tissue sampling.

**Table 1:** Baseline characteristics of HCC patients.

Parameter	Untreated HCC (*n* = 30)	Treated HCC with active tumor cells (*n* = 8)	Treated HCC without active tumor cells (*n* = 12)	Healthy control (*n* = 15)	*p*-value
Male, *n* (%)	27 (90%)	7 (87.5%)	8 (66%)	6 (60%)	0.112
Age, years	66.33 ± 9.11	62.88 ± 10.43	66.17 ± 9.54	35 (19–65)	0.645
Cirrhosis					0.145
Y	18	6	4	N/A	
N	12	2	8	N/A	
Hepatitis					0.820
HBV	4	2	2	N/A	
HCV	8	1	4	N/A	
None	18	5	6	N/A	
TNM stage					**0.023**
T1a–b	10	2	5	N/A	
T2	6	4	0	N/A	
T3–T4	6	2	0	N/A	
TX	8	0	7	N/A	
Tumor size, mm	46.29 ± 42.89	36.88 ± 20.83	35.75 ± 26.63	N/A	0.644
Tumor number					**0.0016**
Solitary	21	2	12	NA	
Multiple	9	6	0	NA	
Albumin, g/L	41.50 ± 6.93	42.25 ± 6.16	44.08 ± 3.13	NA	0.473
Platelets,10^9^/L	194.5 ± 81.88	149.1 ± 62.80	208.1 ± 54.27	NA	0.203
INR	1.083 ± 0.166	1.053 ± 0.067	1.026 ± 0.127	NA	0.539
TB, µmol/L	12.17 ± 7.90	11.63 ± 10.67	9.00 ± 4.11	NA	0.254
AST, IU/L	50.33 ± 31.84	58.57 ± 54.22	33.92 ± 24.95	NA	0.786
Creatinine, g/dl	86.00 ± 28.07	78.88 ± 21.48	88.58 ± 42.47	NA	0.896
TP, mg/dl	74.33 ± 4.57	73.00 ± 7.67	73.83 ± 4.47	NA	0.644
ALP, IU/L	130.6 ± 73.90	141.3 ± 51.17	144.8 ± 151.9	NA	0.473
MELD	8.32 ± 2.74	7.75 ± 1.39	7.72 ± 3.47	NA	0.777

Numbers are presented as mean value ± standard deviation. ALP, alkaline phosphatase; AST, aspartate aminotransferase; HBV, hepatitis B virus; HCV, hepatitis C virus; INR, international normalized ratio; MELD, model for end-stage liver disease; TB, total bilirubin; TNM, tumor (T), nodes (N), and metastases (M); TP, total protein; NA, not available.

Bold values mean lower than 0.05, significant difference.

### Real-Time PCR

Cellular total RNA was isolated using RNeasy Plus Mini kit (Thermo Fisher Scientific, Waltham, USA). The RNA content was determined by measuring the optical density at 260 nm, and cDNA was synthesized according to the instruction described in Prime Scripts RT Master Mix kit. Real-time PCR was performed using SYBRs Premix Ex Taq™ kit (Thermo Fisher Scientific, Waltham, USA). The relative expression of target genes was normalized to GAPDH, analyzed by 2^−ΔΔCt^ method and given as ratio compared with control. Commercial primers for IFN-γ, TNF-α, and IL-17 were purchased from Qiagen (Product No. 249900); primer sequences for other target genes are listed in **Supplementary Table S1**.

### Phenotypic Analysis of PBMCs Using Flow Cytometry

PBMCs were stained for 20 min at 4°C using monoclonal antibodies (mAbs) specific for CD3/Vio-Green, CD4/Alexa Fluor^®^700, CD8/PE-Vio770, CD38/PE-Cyanine5, CD69/APC, PD-1/BV786, CTLA-4/BV421, TIM-3/PE, CXCR3/PE-Vio615, CXCR6/BV711, and CCR5/PE-Vio770. 7-Aminoactinomycin D was used to exclude dead cells from the analysis. Cells were acquired on a 5-laser BD LSRFortessa™ Flow Cytometer. FACS data analysis was performed using FlowJo software (Tree Star Inc., Ashland, USA). The comparisons on the percentages of three T-cell subsets (CD3+CD4+, CD3+CD8+, CD3+CD4-CD8-) were conducted between healthy control and HCC patients (**Supplementary Figure S2** showing the gating strategy for T-cell subsets).

### ELISA

Plasma PD-1 levels were detected using commercial ELISA kits (DuoSet^®^ ELISA Development Systems, Minneapolis, USA) in patients with HCC. Plates were coated overnight at room temperature with capture antibody and then blocked for 1 h at room temperature with 300 µl of filtered 1% BSA in DPBS. For ELISA assays, recombinant PD-1 standards were run with 1:2 serial dilutions. Streptavidin-HRP antibody was used and ELISA plates were developed with SureBlue TMB Microwell Peroxidase Substrate. TMB Stop Solution was added to halt the reaction. The absorbance at 450 nm was measured on a microplate reader (FLUOstar^®^ Omega, BMG Labtech Ltd, Great Britain).

### Statistical Analysis

Statistical analysis was conducted by GraphPad Prism software (version 9.0; GraphPad Software Inc., San Diego, CA). For comparison between two groups, Student’s *t*-test was used while one-way ANOVA was performed for multiple groups. For correlation analysis, linear regression test was carried out. Receiver operator characteristic (ROC) curve was performed to validate diagnostic value. *p*-value <0.05 was considered as statistically different.

## Results

### Data Extraction and Gene Screening

**Figure 1:**
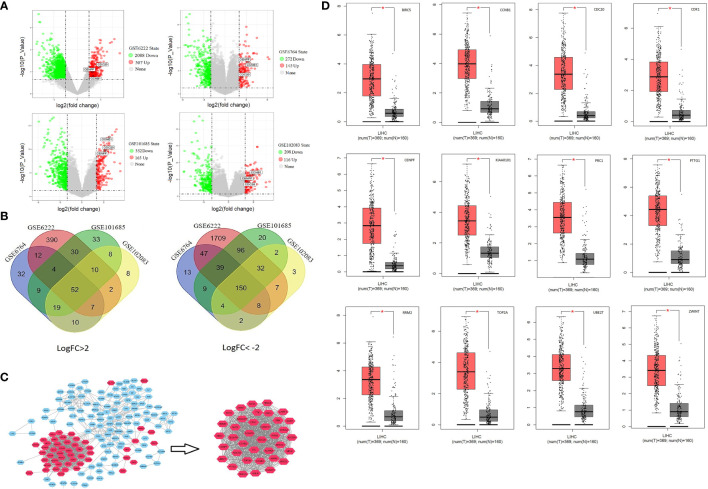
Identify central cluster and gene expression validation: **(A)** Volcano plots of differentially expressed genes. **(B)** Venn Diagram of commonly up-/downregulated genes. **(C)** Screening of central cluster using MCODE plug-in. **(D)** Validation of central cluster genes expression in LIHC (liver hepatocellular carcinoma); red boxplot represents HCC tissue, and black box represents normal liver tissue. *P < 0.05.

The detailed study design is shown in the flow chart (**Supplementary Figure S1**). Of the large number of DEGs, there were 52 upregulated (Log FC >2) and 150 downregulated genes (Log FC < −2) which were found to be commonly expressed in all GSE files (**Figures 1A, B** and **Supplementary Table S2**). The PPI network of DEGs was exported to Cytoscape and 37 central genes were identified through MCODE module analysis (**Figure 1C**). The 37 central genes were further analyzed in the Kaplan–Meier Plotter and GEPIA (**Supplementary Figures S3** and **S4**). A group of 12 genes were identified to be associated with poor prognosis and were significantly upregulated in HCC cancer tissue (**Figure 1D**).

### Enrichment Analysis and Core Gene Selection

KEGG pathway and GO enrichment analysis were performed for the 12 selected genes through ClueGO (GO Term Fusion), Cytoscape. The (KEGG:04115) p53 signaling pathway was found to be the most representative pathway (*p* = 9.60E-6) involved, while negative regulation of mitotic sister chromatid separation (GO: 2000816, *p* = 9.27E-8), chromosome separation (GO:0051304, *p* = 2.18E-8), and mitotic spindle assembly checkpoint (GO:0007094, *p* = 5.97E-6) were the most representative GO terms involved (**Table 2**). CCNB1, CDC20, and CENPF were identified to play an important role in the above pathways and screened as core genes.

**Table 2:** Enrichment analysis of the 12 selected genes using ClueGO plug-in.

GO Term	Term *p*-Value*	Gene Count	Gene ratio	GO Levels	Associated genes found
GO:2000816 negative regulation of mitotic sister chromatid separation	5.55928E-07	4	0.33	[5, 6, 7, 8, 9, 10	[CCNB1, CDC20, CENPF, PTTG1]
GO:0051304 chromosome separation	1.03679E-07	5	0.42	[3, 4]	[CCNB1, CDC20, CENPF, PTTG1, TOP2A]
GO:0007094 mitotic spindle assembly checkpoint	2.09111E-05	4	0.33	[5, 6, 7, 8, 9, 10, 11, 12, 13]	[CCNB1, CDC20, CENPF, PTTG1]
KEGG:04115 p53 signaling pathway	2.87859E-05	4	0.33	[−1]	[CCNB1, CDK1, RRM2, TOP2A]
GO:0030261 chromosome condensation	2.00749E-05	3	0.25	[7]	[CCNB1, CDK1, TOP2A]
GO:0051985 negative regulation of chromosome segregation	1.10394E-06	4	0.33	[3, 4, 5]	[CCNB1, CDC20, CENPF, PTTG1]
GO:1905819 negative regulation of chromosome separation	6.03072E-07	3	0.25	[4, 5, 6, 7]	[CCNB1, CDC20, CENPF]
GO:0051306 mitotic sister chromatid separation	1.60737E-06	3	0.25	[4, 5, 6, 7, 8]	[CCNB1, CDC20, CENPF]
GO:0051784 negative regulation of nuclear division	2.06113E-06	3	0.25	[5, 6, 7]	[CCNB1, CDC20, CENPF]

*Corrected with Bonferroni step down.

### Co-Expression Network of Three Core Genes

**Figure 2:**
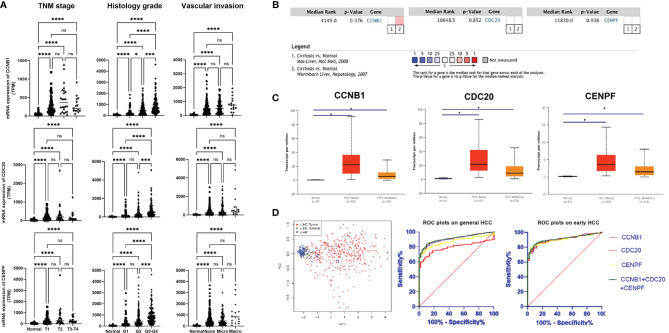
Expression of three core genes across HCC and normal liver tissue: **(A)** Expression of three genes in HCC tumor tissue based on tumor stage, tumor grade, and other classifications. **(B)** Meta-analysis on the expression of three genes in liver tissue based on whether they have cirrhosis or not. **(C)** Expression of three genes in HCC based on whether they have the p53 mutation or not. **(D)** Validation of diagnostic role of three genes using PCA and ROC analysis. Gene expression profiles were downloaded from TCGA database. *P < 0.05, ***P < 0.001, ****P < 0.0001. ns, no significance.

Based on the TCGA data, the expression levels of CCNB1, CDC20, and CENPF varied slightly according to different classifications (TNM staging/Histology Grade/Vascular invasion), but were all upregulated compared with normal liver tissue. With disease progression, their expression levels seemed to increase and showed significant differences from early HCC (T1 stage, G1 grade, none vascular invasion) (**Figure 2A**). In all HCC with p53 mutation, these three genes presented with higher transcription levels. The meta-analysis of two datasets from Oncomine indicated that in comparison with normal liver tissue, the above three genes’ expression demonstrated no statistical differences between normal liver tissue and cirrhotic liver (**Figures 2B, C**).

PCA showed a good diagnostic value using the three core genes. Based on the hepatic expression level of these three core genes, HCC could be clearly distinguished from normal liver tissue and LIHC normal tissue through dimensionality reduction. ROC plots validated that the expression levels of CCNB1 (AUC = 0.905, *p* < 0.001), CDC20 (AUC = 0.793, *p* < 0.0001), and CENPF (AUC = 0.872, *p* < 0.0001) had good diagnostic capabilities in both overall and early-stage (T1) HCC (**Figure 2D**). Further ROC plot also revealed good performance for non-AFP secretor (AFP <7) HCC diagnosis (**Supplementary Figure S7**).

### Expression of Target Genes in Liver Tissue and Blood Samples of Patients

**Figure 3:**
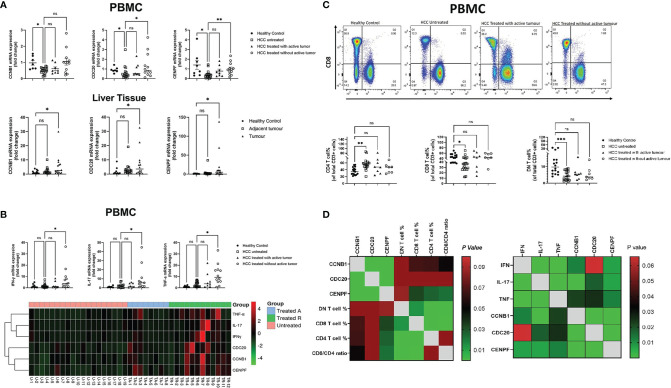
Correlation between core genes and peripheral blood immune cells/pro-inflammation cytokine transcript level: **(A)** PCR results of three genes’ transcript level in HCC PBMCs and liver tissue. **(B)** Transcript level of peripheral blood pro-inflammation cytokines in different subsets of HCC. **(C)** Percentage of three subsets of T cells, CD4+, CD8+, and double-negative (CD4-/CD8-) T cells in different HCC subgroups. **(D)** Correlation matrix of core genes with peripheral blood immune T cells and pro-inflammation cytokine transcript levels *P < 0.05, **P < 0.01, ***P < 0.001. ns, no significance.

To further verify the results obtained from the gene screening, Real-time PCR was conducted using liver tissues and blood samples collected from HCC patients from King’s College Hospital. The expression levels of the three genes were all upregulated in HCC tumor tissue compared with normal liver (*p* = 0.029; *p* = 0.047; *p* = 0.027). In contrast, all three were downregulated in PBMCs from HCC patients (*p* = 0.034; *p* = 0.039; *p* = 0.0132). Furthermore, as the tumor status changed with treatment, the expression levels of these genes changed correspondingly in PBMCs; treated HCC patients without active/residual tumor cells had higher transcript levels of CCNB1, CDC20, and CENPF compared with the other two subgroups of HCC patients (**Figure 3A**).

### Cytokine Gene Expression and Plasma PD-1 Levels

Treated HCC patients without active/residual tumor cells had higher mRNA expression levels of antitumor cytokines, including IFN-γ, TNF-α, and IL-17 when compared with untreated HCC patients (*p* = 0.016; *p* = 0.041; *p* = 0.0146). With tumor response to treatment, the transcript levels of these genes further increased (**Figure 3B**). Soluble PD-1 levels in plasma measured by ELISA were not significantly different between patients with untreated and treated HCC with active/residual tumor cells. However, untreated HCC patients had higher plasma PD-1 levels compared with healthy controls (*p* < 0.05) and treated HCC patients without active/residual tumor cells (*p* < 0.01) (**Figure 3B**).

### T Cell Profiling

The percentages of peripheral blood T cell subsets were defined by flow cytometry and showed significant differences between untreated HCC patients and healthy controls. The percentages of both CD8+ and double-negative (CD8-CD4-) T cells were lower in untreated HCC patients (*p* = 0.01; *p* = 0.0009), while the portion of CD4+ T cells was higher in untreated HCC (*p* = 0.0014) (**Figure 3C**) compared with healthy controls. No difference was found in the percentage of cells positive for exhaustion/activation markers (CD38, CD69, CTLA-4, Tim-3, and PD-1). High levels of expression of CXCR3 and CCR5 were observed in peripheral blood T cells, although there was no difference between the three HCC untreated or treated subsets (**Supplementary Figure S5**).

### Correlations Between the Three Target Genes and Immunity

Correlation matrix revealed that the expression levels of CCNB1, CDC20, and CENPF were all correlated with CD8+ and CD4+ T cells to an extent, but only CENPF showed statistical significance (*p* = 0.033; *p* = 0.026). Though CCNB1 showed a trend, no significance was observed (*p* = 0.067; *p* = 0.065). In addition, the three genes expressed positive association with antitumor cytokine mRNA expression level in PBMCs (**Figure 3D**).

**Figure 4:**
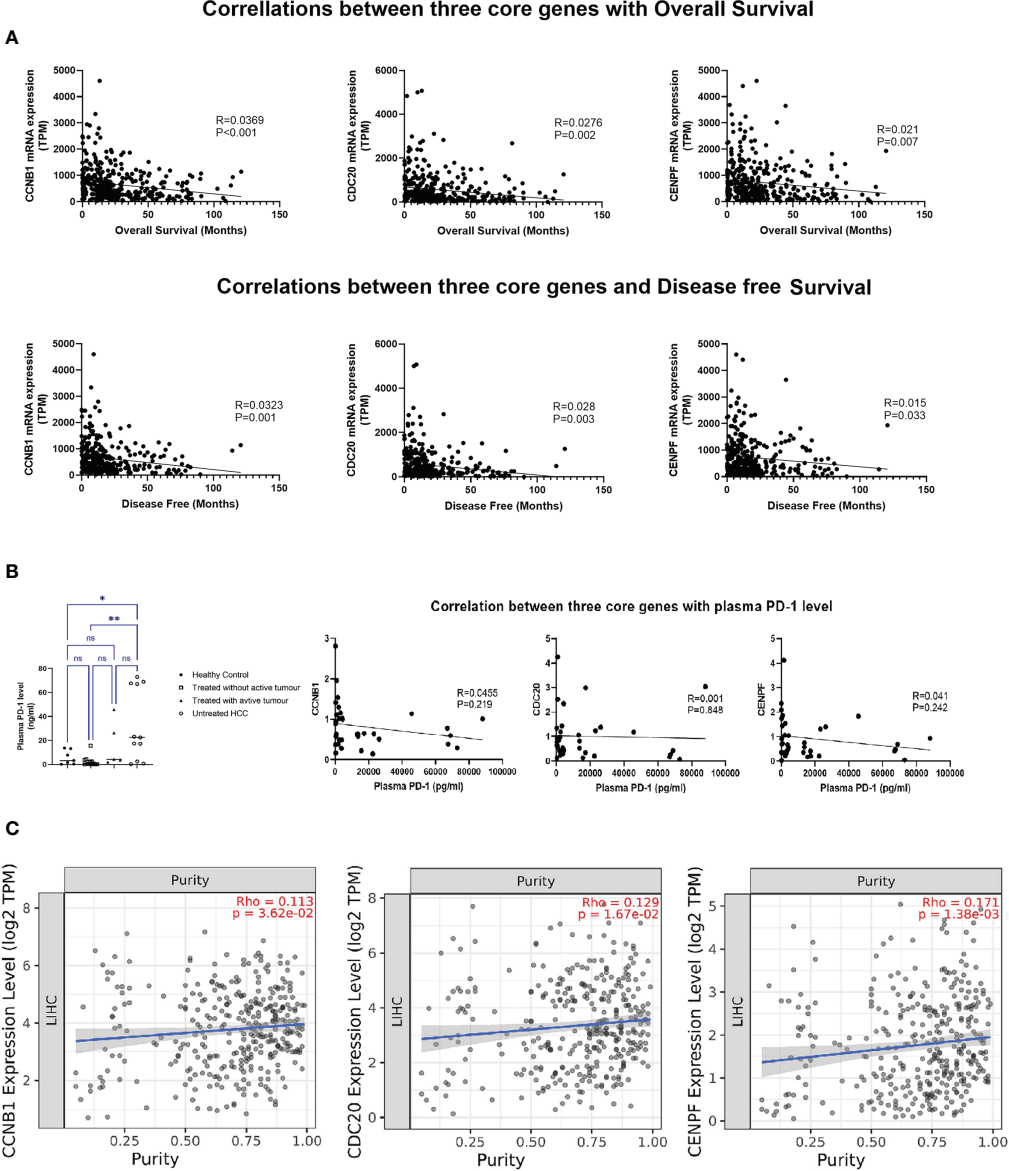
Correlation analysis of three core genes: **(A)** Correlation with overall survival and disease-free survival. **(B)** Correlation with plasma PD-1 level. **(C)** Correlation with tumor purity in HCC. *P < 0.05, **P < 0.01. ns, no significance.

Increasing expression of these three genes in HCC tumor tissue was associated with decreased overall and disease-free survival (**Figure 4A**). Though the expression levels of three core genes in PBMCs illustrated no correlation with the plasma PD-1 levels (**Figure 4B**), in HCC tumor tissue, all of them were positively associated with tumor purity (percentage of tumor cells within HCC tissue); the higher the expression in HCC tissue, the more tumor cells were present in the tissue with fewer infiltrating immune cells (**Figure 4C**). Moreover, a significant association between the transcript levels of these three genes and inhibitory checkpoint molecules was observed in the tumor environment (**Supplementary Figure S6**).

## Discussion

In this study, joint screening of multiple databases including GEO, TCGA, GEPIA, and Kaplan–Meier Plotter identified CCNB1, CDC20, and CENPF, three mutated gene markers. These three genes were found to be widely expressed and highly upregulated in HCC samples, and their upregulation was significantly associated with poor prognosis. By using meta-analysis through the Oncomine database, the influence of liver cirrhosis on the expression of above three genes was excluded. Our findings may be useful in tackling the difficulties caused by the heterogeneity of HCC, which contributes to the failure of precision therapy with targeted drugs (21–23). The differential expression of various genes in the tumor tissue makes it unlikely that a single treatment will be effective in all patients. For example, although anti-PD1 therapy is an effective cancer treatment, the overexpression or mutations of LAMA3, CXCR2, and JAK1/2 could prevent the immune system from boosting effective antitumor immunity (24, 25). Therefore, identifying genes that are commonly upregulated in HCC tissue may provide new targets for HCC treatment.

Enrichment analysis revealed that the above three genes mainly interact with each other in functions related to cell proliferation and cell cycle (**Table 2**). It is known that one of the distinguishing features of cancer is the dysregulation of the cell cycle, resulting in unrestricted proliferation of cancer cells (26, 27). Normally, p53 gene regulates all four checkpoints in the cell cycle (G1/S; S-phase; G2/M; M) (28, 29). It can halt the cell cycle when censored DNA damage or other gene mutation occurs, thereby encouraging repair of damaged DNA (30). However, in patients with HCC, the p53 gene has always presented with a degree of loss of function or dysfunction (31), resulting in cancer cells continually proliferating leading to tumor growth. The results of our investigation showed that the expression of CCNB1, CDC20, and CENPF are closely related to p53 mutations and the p53 signaling pathway (**Table 2**). In HCC tissue expressing the p53 mutation, the expression levels of the three genes are significantly higher than those without p53 mutator (**Figure 2C**). Meanwhile, it will also increase with the upgrading of TNM stage/histological grade and the deepening of vascular invasion degree (**Figure 1**). Thus, overexpression of CCNB1, CDC20, and CENPF in liver tissues imply an underlying cell cycle disorder. The higher the expression, the faster the proliferation of abnormal cells and the greater the possibility of tumor development. This was also confirmed in other solid tumors, not only in HCC. Fang et al. found that downregulation of CCNB1 impaired colorectal cancer proliferation *in vitro* and tumor growth *in vivo* (32). Through a long-term follow up, Karra et al. reported that CDC20 and securin overexpression predict short-term breast cancer survival (33). Han et al. also found that CENPF could promote papillary thyroid cancer progression by mediating cell proliferation and apoptosis (34).

Early stage of HCC is usually asymptomatic, and the guidelines of American Association for the Study of Liver Diseases (AASLD) and European Association for the Study of the Liver (EASL) recommend imaging (ultrasound, CT or MRI) combined with alpha-fetoprotein (AFP) to identify HCC (35–37). However, the sensitivity and specificity of AFP in the diagnosis of liver cancer is not ideal. The specificity of AFP is low and it can be elevated in pregnant women, acute and chronic hepatitis, gonadal tumors, and gastrointestinal tumors; in addition, approximately 40% of patients with HCC are non-secretors of AFP (38–40). Therefore, it is meaningful to identify new markers for HCC diagnosis. Some studies reported that YTH N6-methyladenosine RNA binding protein 1 (YTHDF1) and DNA primase subunit 1 (PRIM1) might be potential molecular targets for HCC (41, 42). Those two molecular targets regulated the proliferation, migration, and invasion of HCC cells. In the present study, we aim to identify key genes in HCC that are associated with antitumor immunity and also the pathways influencing the antitumor immunity, such as inhibitory checkpoints. Regardless of the classification standards (TNM staging/Histology Grade/Vascular invasion) used for analysis, all results revealed that CCNB1, CDC20, and CENPF were already significantly upregulated in the early stages of HCC tumor tissues (T1/G1/Stage I/no vascular invasion). There might be a limitation due to the fact that the final reading for RNA-seq is from the mean value of several reads at the same tumor site rather than the average value from different tumor sites. Further ROC analysis showed that these three genes have high value for the early diagnosis of HCC Moreover, even in non-AFP secretors, AUCs of CCNB1, CDC20, and CENPF can achieve a performance close to 0.90 (**Supplementary Figure S7**). Considering that genetic changes often occur earlier than clinical/pathological changes, the combination of serum AFP and tumor-related gene expression may enhance the efficacy of screening and early diagnosis of HCC (43–45).

Though the RNA-seq data from TCGA did not have detailed background information for patients’ underlying liver diseases, we found that the expression levels of the three genes were closely related to the survival of patients with HCC. Tumor immune infiltrate analysis through the TIMER database demonstrated that the expression levels of the three genes were proportional to the tumor purity. As mentioned above, due to the close relationship between CCNB1, CDC20, and CENPF and p53, overexpression of these three genes represents an imbalance in cell cycle regulation. In HCC, it indicates that cancer cells are rapidly proliferating, and the tumor is in an aggressive state. This feature could be used to assess the prognosis of HCC patients after surgery, to indicate the risk of recurrence, to stratify follow-up imaging, and to guide clinicians regarding adjuvant treatment.

Intriguingly, the expression levels of CCNB1, CDC20, and CENPE in PBMCs were completely opposite to those observed in tumor tissues. Compared with healthy controls, the expression of the three genes in PBMCs from untreated HCC was significantly downregulated, and with treatment, the expression levels in PBMCs gradually increased. The discrepancy in expression pattern between peripheral blood and liver tissue prompted us to set up the correlation network of three target genes in the peripheral blood with immune cells and antitumor cytokines, which showed that the three target genes not only correlated with the percentage of T-cell subtypes, but also showed a positive correlation with the transcription levels of antitumor cytokines (IFN-γ, TNF-α, and IL-17). It can be speculated that the discrepancy in the expressions of CCNB1, CDC20, and CENPF between tumor tissue and peripheral blood may be related to differences in the immune environment: the peripheral circulation has higher percentage of T effectors, such as CD8+T cells, representing cytotoxic T cells, NK cells, and other anti-tumor immune cells than that in HCC tissue where it was dominated by cancer cells and tumor-promoting lymphocytes. Therefore, the overall microenvironment in peripheral blood often presents an immune state of promoting antitumor activity while the HCC tissue shows a state of immunosuppression. The different immune status of peripheral blood and tumor tissue could also explain why the expression levels of the three genes in PBMCs have no correlation with the soluble PD-1 level, while in HCC, all of them were strongly associated with the mRNA expression of immune checkpoint molecules: PD-1, CTLA-4, and TIM-3. The close association between these three genes and p53 as well as inhibitory checkpoint molecules suggests that those genes are potentially treatment targets for HCC. The transcription level changes of the three genes in peripheral blood may assist in establishing rapid, reliable, and reproducible detection assays to assess the immune status and treatment response of HCC patients.

Given the fact that the mRNA expression of three target genes in PBMCs was opposite to that in tumor tissues, single-cell omics and spatial transcriptomics might be applied in future studies. The establishment of new models and algorithms (46, 47) allows information from various single-cell omics databases to be mutually compatible and integrated even though the experimental conditions, platforms, and omics types are different. Meanwhile, high-throughput spatial transcriptomics makes it possible to measure all genetic activity in the tissue samples and to locate the position of the activity. It looks encouraging that more novel methods have been developed to deconvolute spatial transcriptomics for decomposition of cell mixtures in the spatially resolved transcriptomics data (48, 49). Moreover, combining single-cell RNA-seq and spatial transcriptomics could be a promising new method to comprehensively analyze the spatial cell composition of tumor tissue, to characterize tumor cells and their immune microenvironment, and, more importantly, to define the interactions between tumor and microenvironment. Such applications have been found in cancer studies other than HCC (50, 51). Further integrative analysis by using both single-cell omics and spatial transcriptomics might provide a more comprehensive understanding about the cellular process in HCC development.

In conclusion, we confirmed that the three genes, CCNB1, CDC20, and CENPF, are commonly involved in the carcinogenesis of HCC and showed potential for early diagnosis. More importantly, the expression of these three genes is closely associated with Th1/Th17 cytokine gene expression and CD4^pos^/CD8^pos^ T-cell percentage in peripheral blood and inhibitory checkpoints in tumor microenvironment.

## Data Availability Statement

The original contributions presented in the study are included in the article/**Supplementary Material**. Further inquiries can be directed to the corresponding authors.

## Ethics Statement

The studies involving human participants were reviewed and approved by Ethics Committee of King’s College Hospital. The patients/participants provided their written informed consent to participate in this study.

## Author Contributions

Study design: TS, ZH, and YM. Manuscript writing: TS, ZH, YJ, AW-J, SG, and MH. Data analysis: TS, YJ, AW-J, SG, MH, and RH. Result discussion: TS, ZH, YJ, SG, MH, RH, and YM. Proofreading and editing: SK, WJ, NH, and YM. All authors contributed to the article and approved the submitted version.

## Funding

TS is supported by State Scholarship Fund (201808310051), China Scholarship Council; The Henry Lester Trust; and Surgical Funds, King’s College Hospital Charity. ZH is supported by the Visiting Scholar Funding from Shanghai University of Traditional Chinese Medicine, China.

## Conflict of Interest

The authors declare that the research was conducted in the absence of any commercial or financial relationships that could be construed as a potential conflict of interest.

## Publisher’s Note

All claims expressed in this article are solely those of the authors and do not necessarily represent those of their affiliated organizations, or those of the publisher, the editors and the reviewers. Any product that may be evaluated in this article, or claim that may be made by its manufacturer, is not guaranteed or endorsed by the publisher.
