# Supplementary figures and images for: Expression Levels of Three Key Genes CCNB1, CDC20, and CENPF in HCC Are Associated With Antitumor Immunity
Source: Front Oncol. 2021 Sep 30;11:738841. doi: 10.3389/fonc.2021.738841 (PMC8515852; doi:10.3389/fonc.2021.738841)

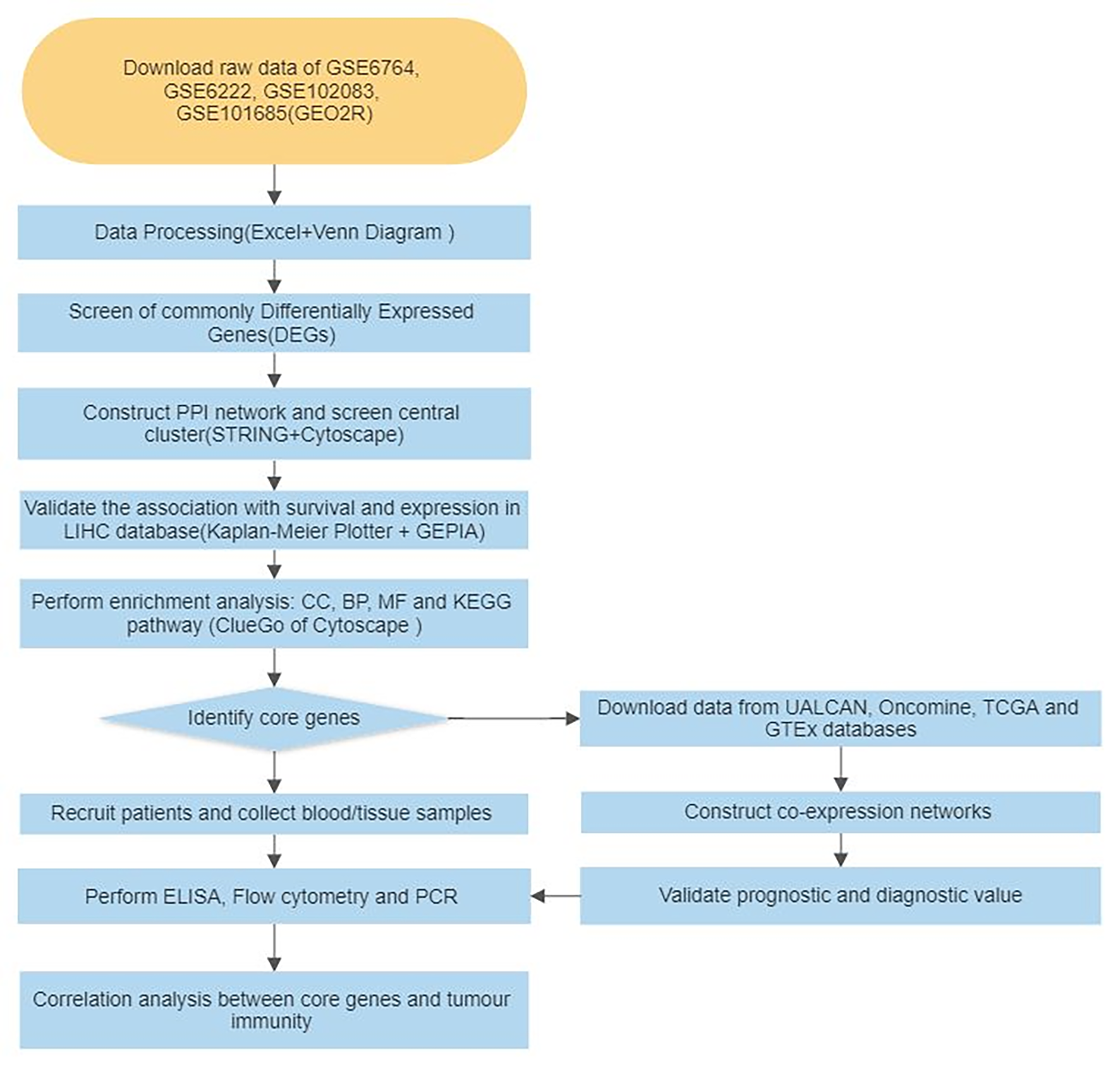

Supplement: Supplementary file 1 [file Image_1.tif]

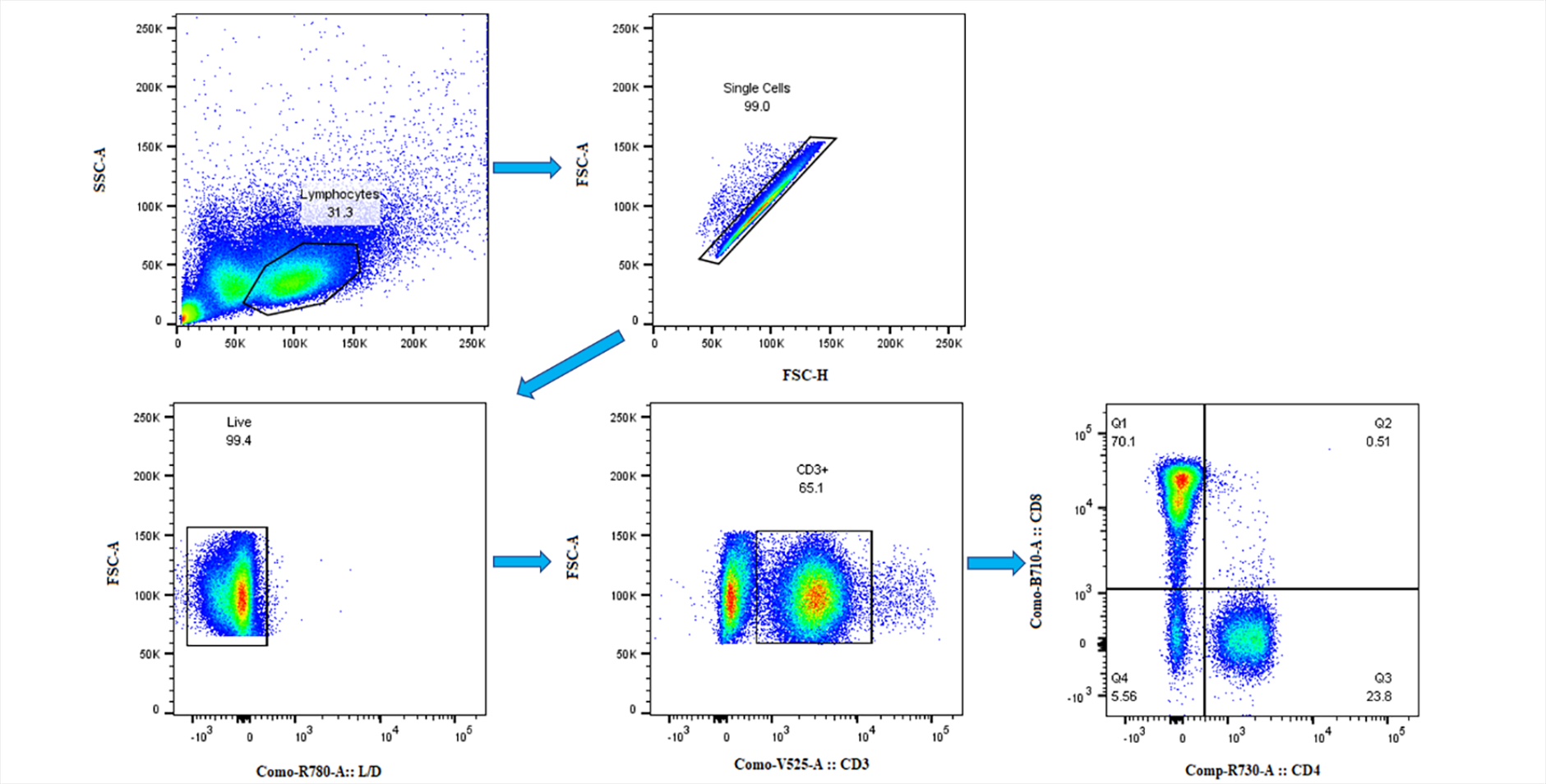

Supplement: Supplementary file 2 [file Image_2.tif]

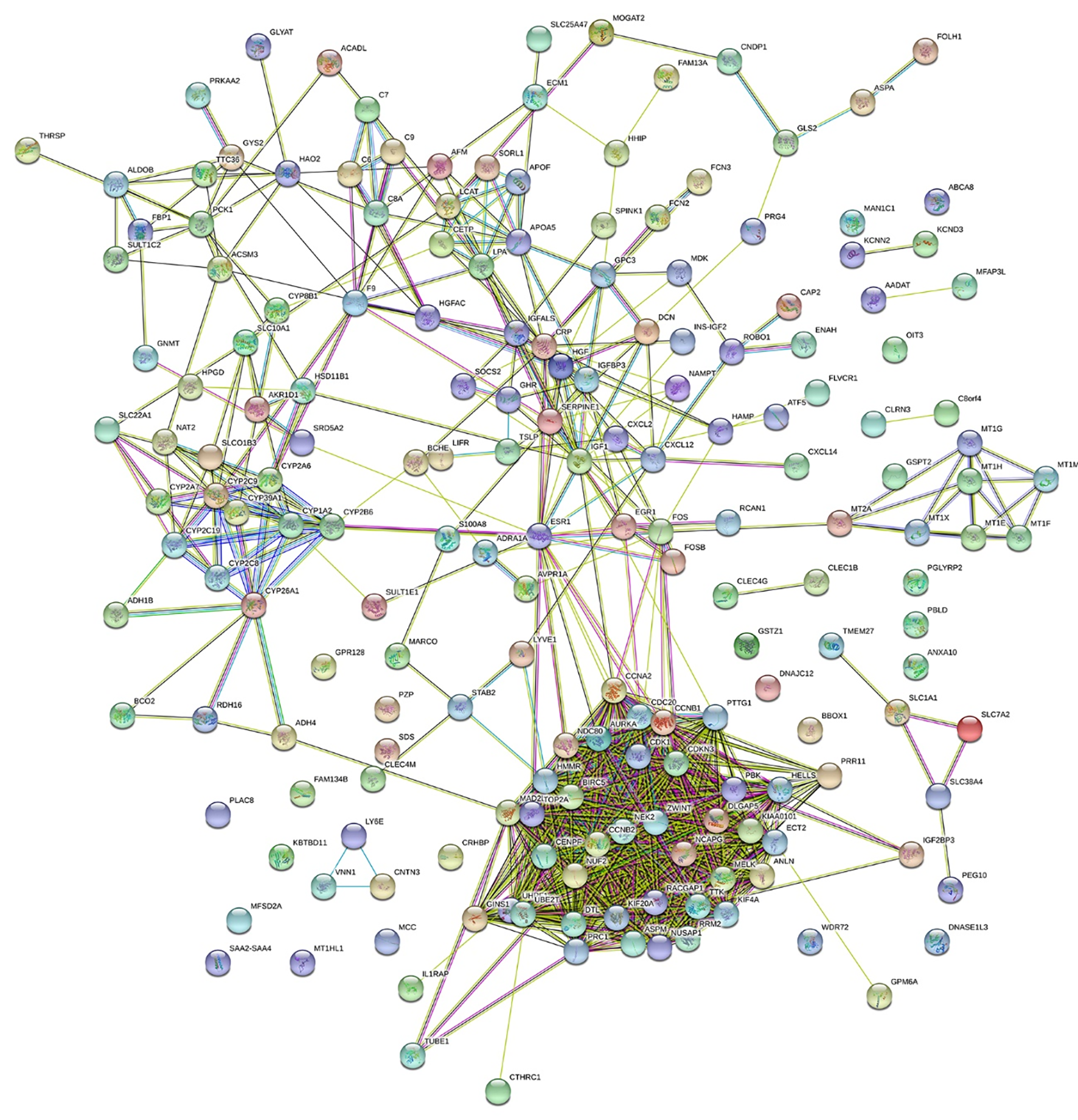

Supplement: Supplementary file 3 [file Image_3.tif]

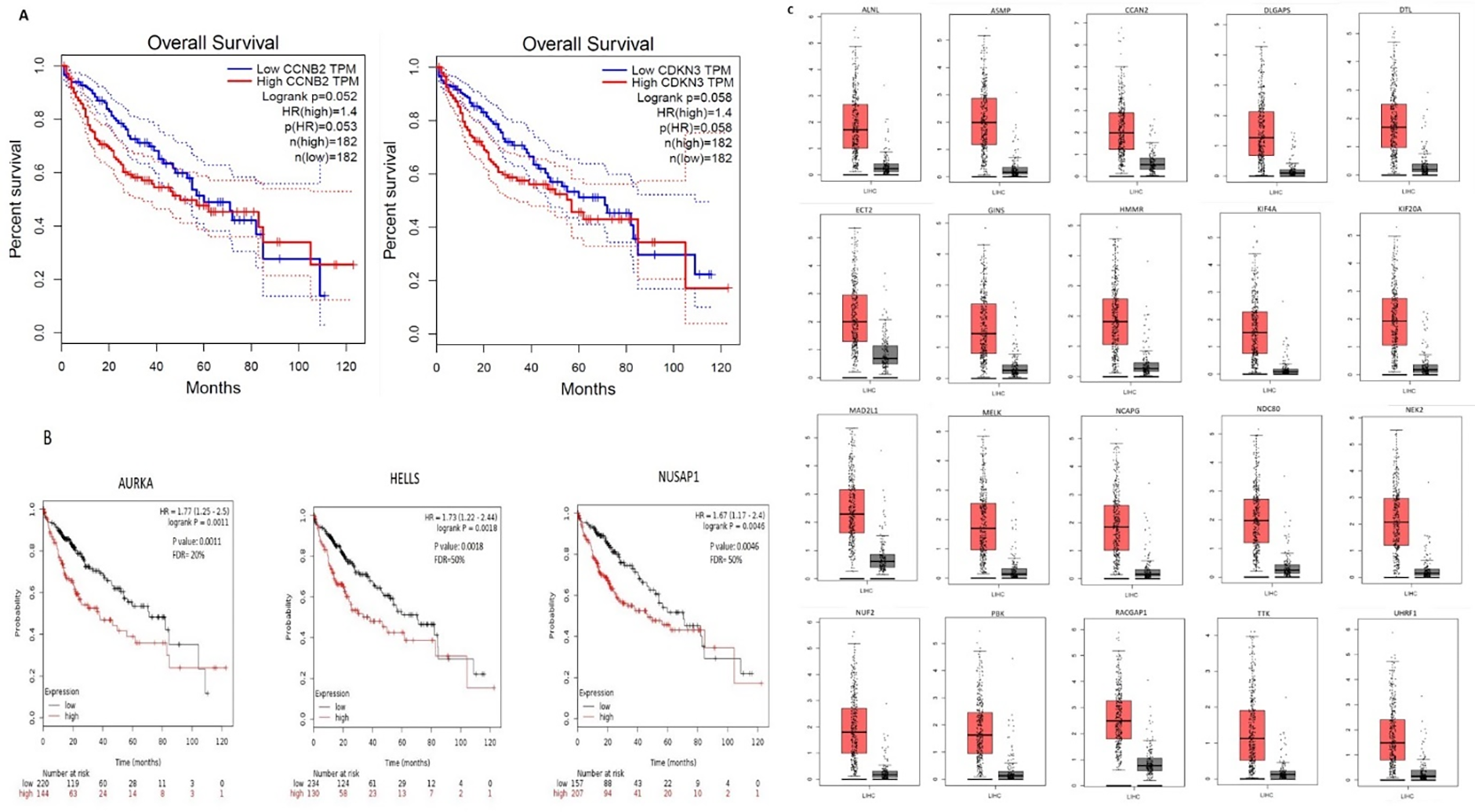

Supplement: Supplementary file 4 [file Image_4.tif]

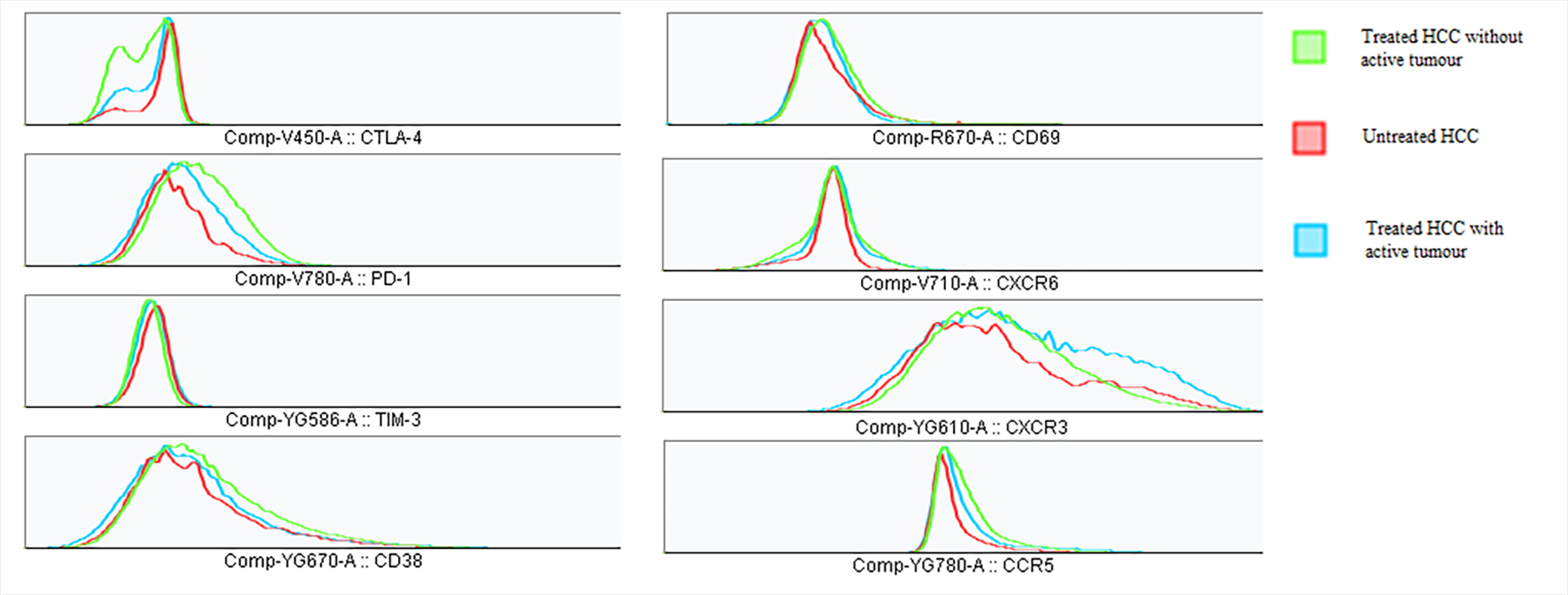

Supplement: Supplementary file 5 [file Image_5.tif]

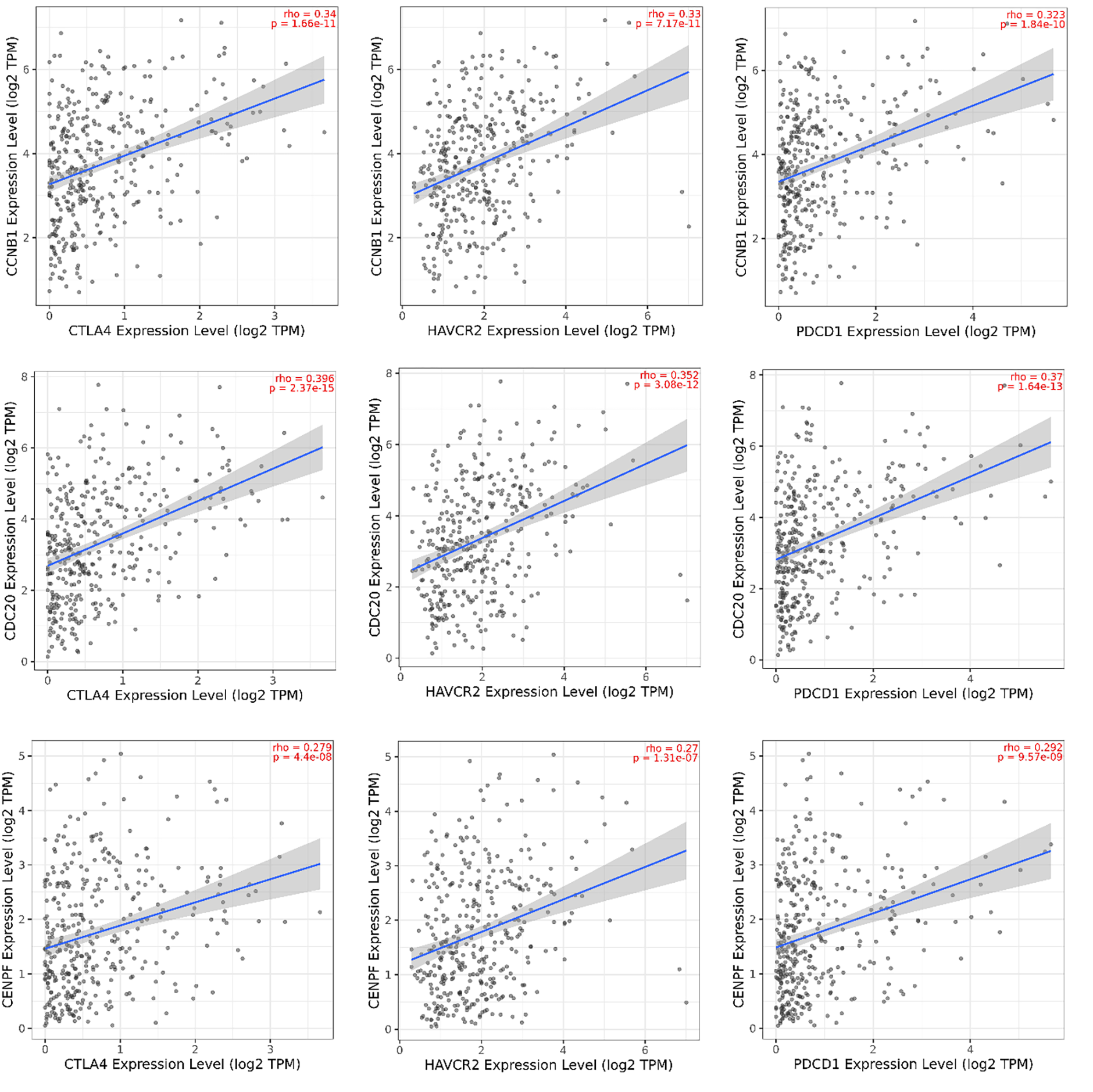

Supplement: Supplementary file 6 [file Image_6.tif]

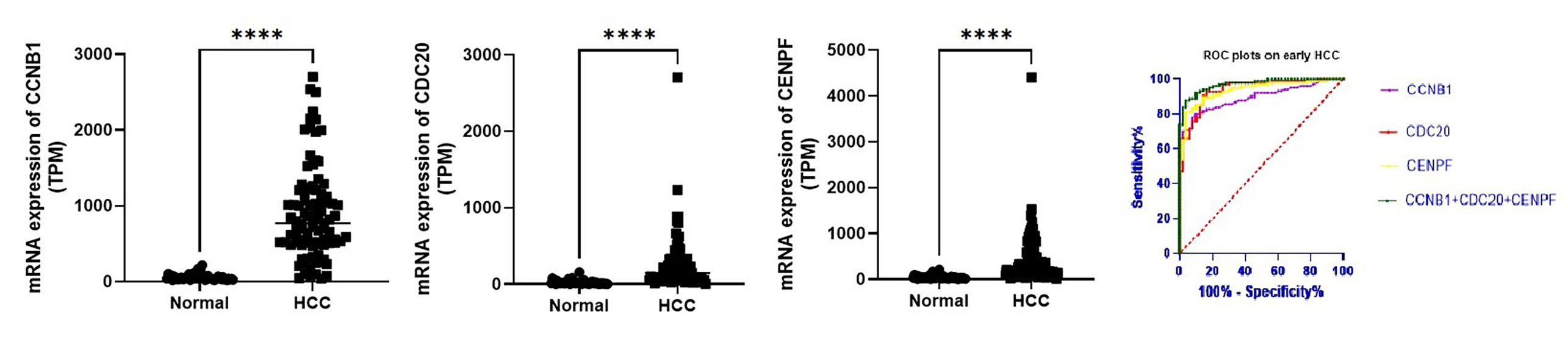

Supplement: Supplementary file 7 [file Image_7.jpeg]

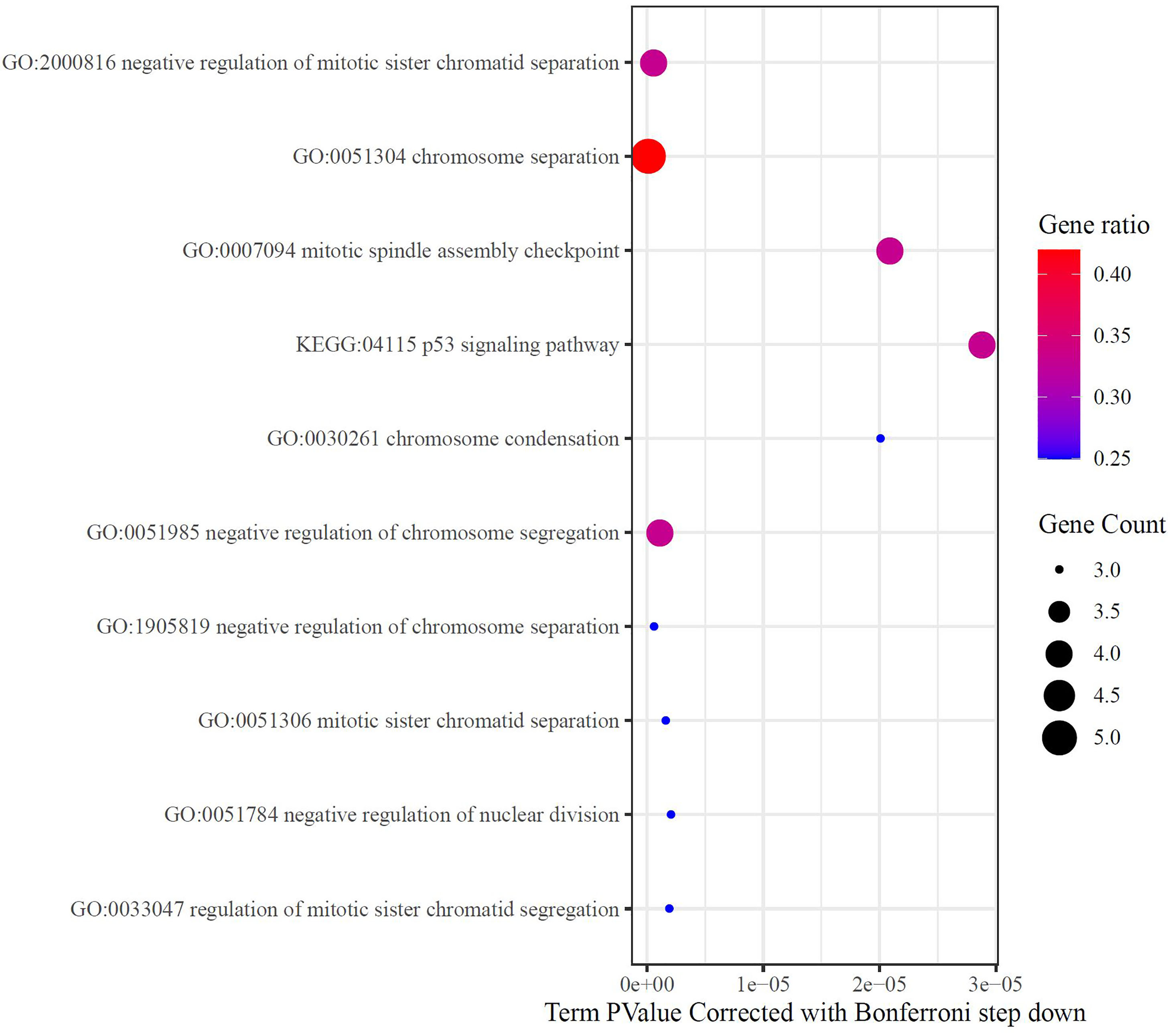

Supplement: Supplementary file 8 [file Image_8.tif]
