# Supplementary material for: Expression Levels of Three Key Genes CCNB1, CDC20, and CENPF in HCC Are Associated With Antitumor Immunity
Source: Front Oncol. 2021 Sep 30;11:738841. doi: 10.3389/fonc.2021.738841 (PMC8515852; doi:10.3389/fonc.2021.738841)
Supplement: Supplementary file 9 [file Table_1.docx]

Supplementary Table 1. List of Primers for Real-time PCR

| Target | Primer | Sequence (5'-3') |
| --- | --- | --- |
| CCNB1 | FP | GGCCTCTACCTTTGCACTTCCT |
|  | RP | GCTCGACATCAACCTCTCCAA |
| CENPF | FP | CGCCAGAACTGTACTCTCCG |
|  | RP | GTAGGCAGCCCTTCTTTCCA |
| CDC20 | FP | GCTTTGAACCTGAACGGTTTTG |
|  | RP | TCTGGCGCATTTTGTGGTTTT |
| GAPDH | FP | ACAACTTTGGTATCGTGGAAGG |
|  | RP | GCCATCACGCCACAGTTTC |

FP, Forward Primer; RP, Reverse Primer

Supplementary Table 2. Common differentially expressed genes (DEGs) among 4 gene chips, including 52 up-regulated genes and 150 down-regulated genes (|Log FC|>2) in HCC tissue compared with normal liver tissue.

| DEGs | Gene Symbol |
| --- | --- |
| Upregulated | SPINK1 PRKAA2 IGF2BP3 CCNB1 ASPM FLVCR1 GINS1 ANLN BIRC5 CCNB2 PRC1 CDK1 RACGAP1 RRM2 MDK TOP2A HELLS SULT1C2 ENAH AURKA MAD2L1 DLGAP5 UBD///GABBR1 CAP2 DTL HMMR KIF4A GPC3 ROBO1 KIF20A MELK PEG10 CDC20 CTHRC1 UHRF1 ZWINT NDC80 KIAA0101 CCNA2 TTK CDKN3 PBK NUF2 NCAPG PRR11 PTTG1 NEK2 UBE2T DUXAP10 CENPF NUSAP1 ECT2 |
|  |  |
|  |  |
| Down regulated | CYP26A1 BBOX1 IGF1 CYP39A1 FAM134B SORL1 ACSM3 CYP2C19 GHR CLEC1B BCHE CRHBP SLC38A4 ACADL CRP SRD5A2 ADRA1A PBLD MT1G LINC01093 KCND3 RCAN1 CYP2C9 CYP2A7 CLEC4M ESR1 MFAP3L GPM6A MAGI2-AS3 CYP2B7P///CYP2B6 APOF FAM13A SDS LINC01554 SLC10A1 AKR1D1 HGF C7 NAMPT INS-IGF2///IGF2 MT1E ANXA10 TTC36 GYS2 CXCL2 LYVE1 NAT2 FBP1 ADH4 OIT3 MCC CETP MT1HL1 TSLP HAO2 MT1F KCNN2 GSTZ1 CNDP1 FOSB MARCO LCAT IGFBP3 HAMP DCN CYP8B1 STAB2 HGFAC MT2A PZP GLS2 C6 PLGLB1///PLGLB2 C9 CXCL14 C8A SLCO1B3 BCO2 C3P1 FOLH1B DNASE1L3 C8orf4 F9 IGFALS MT1M EGR1 ECM1 FCN2 CYP2A6 ABCA8 PGLYRP2 SLC22A1 MFSD2A APOA5 ASPA MAN1C1 SAA2-SAA4///SAA4 LOC100287413///GLYATL1 PCK1 KBTBD11 ATF5 IL1RAP RDH16 TMEM27 AFM HPGD DNAJC12 THRSP MT1X S100A8 AADAT MOGAT2 SLC7A2 LINC00844 TUBE1 CNTN3 SULT1E1 SLC25A47 FLJ22763 PRG4 CYP1A2 LPA GSPT2 LIFR VNN1 SLC1A1 FOS CXCL12 GNMT AVPR1A WDR72 GLYAT SERPINE1 SOCS2 CYP2C8 HHIP ADH1B LY6E FOLH1B///FOLH1 FCN3 GBA3 CLEC4G CYP2B6 CLRN3 MT1H HSD11B1 LOC101928916///NNMT PLAC8 ALDOB ADGRG7 |

Supplementary Table 3 Flow cytometry panel for T cells

| Antigen | Colour | Laser Channel | Purpose | CAT# |
| --- | --- | --- | --- | --- |
| Live/Dead | APC-Cy7 | R780 | Live cells | 423101 |
| CD3 | Vio-Green | V525 | T cell differentiation | 5170601466 |
| CD4 | AlexaFlour700 | R730 | T cell lineage | 7052566 |
| CD8 | PerCP-Vio770 | B710 | T cell lineage | 5180517917 |
| CD38 | PE-Cy5 | YG670 | Activation | 7188703 |
| CD69 | APC | R670 | Activation | 5191018365 |
| CCR5 | PE-Vio770 | YG780 | differentiation | 5170606133 |
| CXCR3 | PE-CF594 | YG610 | differentiation | 9031960 |
| CXCR6 | BV711 | V710 | differentiation | 932039 |
| PD-1 | BV786 | V780 | Exhaustion | 563789 |
| CTLA-4 | BV421 | V450 | Exhaustion | 369606 |
| TIM-3 | PE | YG586 | Exhaustion | 5170606143 |
